# Supplementary material for: CCK Receptor Inhibition Reduces Pancreatic Tumor Fibrosis and Promotes Nanoparticle Delivery
Source: Biomedicines. 2024 May 7;12(5):1024. doi: 10.3390/biomedicines12051024 (PMC11118934; doi:10.3390/biomedicines12051024)
Supplement: Supplementary file 1 [file biomedicines-12-01024-s001.zip › biomedicines-2959503-supplementary.pdf]

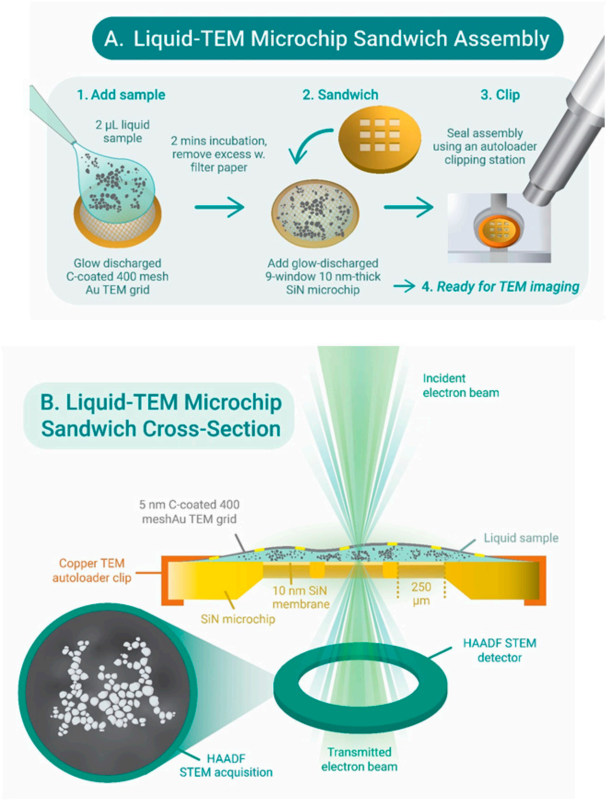

Figure S1. Schematic of liquid-TEM microchip sandwich enclosure used to assess NanoJackets. (A). Liquid-TEM microchip sandwich assembly used to make the liquid cell, where (1) a 2 µL liquid sample was deposited onto a glow discharged 5 nm-thick carbon-coated 400 mesh gold TEM grid. After a 2-minute incubation, excess liquid was removed with filter paper and then (2) sandwiched by a glow-discharged 9-window 10 nm-thick silicon nitride microchip and finally (3) sealed using an autoloader grid clip. (B). The cross-section of the liquid-TEM microchip sandwich assembly highlights the configuration of the liquid cell with the encapsulated thin liquid layer and membrane thicknesses. High-angle annular dark-field imaging (HAADF) STEM imaging methodology is shown, where a higher intensity nano-probe electron beam is rostered and high angle, incoherently scattered electrons are detected through an annular HAADF detector to form an image.

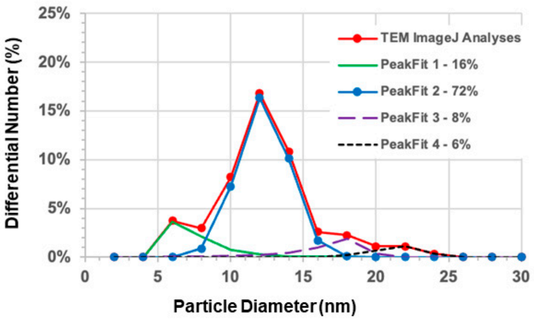

Fitted Parameters for Log Normal Diameter Distribution

r^2 Coef Det	DF Adj r2	Fit Std Err	F-value
0.99052942	0.93370591	0.01031536	28.5245763

Peak	Type	a_0	a_1	a_2
1	Log Normal Area	0.161	6	0.266
2	Log Normal Area	0.717	12	0.140
3	Log Normal Area	0.086	18	0.0957
4	Log Normal Area	0.0402	22	0.0731

Figure S2. Particle size diameter determined using NIH ImageJ digital analyses of liquid cell STEM photomicrographs. (Left) A total of 134 nanoparticles evaluated in 6 different liquid cell STEM photomicrographs. There were four peaks deconvoluted from the measured particle diameter distribution. Each diameter bin is 2 nm wide. (Right) The four distributions deconvoluted from the experimental data centered via log normal diameter distributions at 6 nm 16%of peak area), 12 nm

(72% of peak area), 18 nm (8% of peak area), and 22 nm (4% of peak area. The r^2 value for the calculated fit to the experimental data is 99.05%. Most of the log normal particle diameter distribution (72 percent based on area) is centered at 12 nm with the log normal standard deviation at sz = 0.266. There was no correction made for baseline.
